# Rural-to-urban migration, discrimination experience, and health in China: Evidence from propensity score analysis

**DOI:** 10.1371/journal.pone.0244441

**Published:** 2020-12-28

**Authors:** Zihong Deng, Yik Wa Law

**Affiliations:** Department of Social Work and Social Administration, The University of Hong Kong, Hong Kong SAR, China; Catholic University of Korea College of Medicine, REPUBLIC OF KOREA

## Abstract

This research examines how rural-to-urban migration influences health through discrimination experience in China after considering migration selection bias. We conducted propensity score matching (PSM) to obtain a matched group of rural residents and rural-to-urban migrants with a similar probability of migrating from rural to urban areas using data from the 2014 China Family Panel Studies (CFPS). Regression and mediation analyses were performed after PSM. The results of regression analysis after PSM indicated that rural-to-urban migrants reported more discrimination experience than rural residents, and those of mediation analysis revealed discrimination experience to exert negative indirect effects on the associations between rural-to-urban migration and three measures of health: self-reported health, psychological distress, and physical discomfort. Sensitivity analysis using different calipers yielded similar results. Relevant policies and practices are required to respond to the unfair treatment and discrimination experienced by this migrant population.

## Introduction

Given the large scale of and rapid increase in migration worldwide in recent years, the study of the relationship among migration, discrimination, and health is highly pertinent to promoting migrant well-being. China, in particular, has experienced unprecedented internal migration, with the number of migrants, most of them rural-to-urban migrants, increasing from 6.57 million in 1982 to 221.43 million in 2010, with the annual increase rate of around 10% from 2005 to 2010 [1]. Data released by the National Bureau of Statistics (2012–2019) show, however, that the growth in the migrant population slowed from 2010 to 2014, and, since 2015, the size of that population has actually decreased [2]. Migrants in China are confronted with many difficulties, not least discrimination and health problems, with research revealing greater health depletion the longer migrants remain in the migration destination [3, 4]. The extant literature also shows discrimination experience to be associated with poorer health, including self-rated physical health and depressive distress [5, 6], which may help to explain the health disparities between migrants and non-migrants. The influence of discrimination experience on health has been studied in a large body of literature focusing on international migrants and racial/ethnic minority groups, and some studies in China have also examined how discrimination influences health among migrants [5, 7], but the role of such experience in the relationship between rural-to-urban migration and health in China remains unclear. Indeed, rural-to-urban migrants in China are likely to have considerable exposure to unfair treatment and discrimination, in part because of the rural-urban gap and various institutional barriers during the migration process [8, 9]. Therefore, it is of interest to examine how rural-to-urban migration influences discrimination experience and further influences health outcomes. In addition, rural-to-urban migrants are a self-selected group, with some individuals more likely than others to migrate from rural to urban areas. The factors affecting the migration decision may further influence post-migration discrimination experience and health, and it is thus necessary to address the aforementioned self-selection bias before conducting further analysis.

### Migration and health status

Migration is considered to be an important factor influencing health outcomes. The relationship between migration and health is complicated through the immediate and offsetting pathways, with some factors harmful and others beneficial for health, including both physical health and mental health [10, 11]. The extant literature reports mixed results of health differences between migrants and non-migrants. A group of studies reveals that migration is positively associated with health, or the association between migration and health is not significant. The positive effect of migration on health is usually related to the increase of income after migration and better health services and resources in the migration destination. Researchers found that after controlling the factors that affect the migration decision and health consequences in propensity score matching (PSM), short-term migration positively influence self-reported health. They also documented a close to zero impact of long-term migration on self-reported health in a panel dataset in China using rural non-migrants as the comparison group [12]. In terms of mental health, rural-to-urban migrant workers do not constitute a more vulnerable group than rural or urban residents, possibly due to the facts that the higher economic status and life opportunities that most migrants enjoy may lead to better mental health [13]. Zhang et al. [14] also revealed that migration status was not significantly associated with psychological health using nationally representative data in China [14].

It has been argued that migration can be a stressful situation during which migrants’ health may deteriorate over time in the destination areas. In China, rural-to-urban migrants reported suffering a worse mental health status than both urban residents in immigrant communities and their rural counterparts in emigrating communities [15]. A literature review summarizes that major depression, depressive symptoms, and insomnia are the most common psychological outcomes among Chinese migrants and their families [16]. Migration and its related factors, such as subjective and objective socioeconomic status, social support, adaptation and difficulties adjusting to a new environment, duration of migrant status, and social stigma, are associated with poor mental health [17, 18].

With respect to physical health, rural-to-urban migration is considered to be the main demographic factor driving the cardiovascular disease epidemic in China [19], and data from other countries also support the link between such migration and such cardiovascular risk factors as obesity, hypertension, and diabetes, with the impact of these risk factors associated with the age at which migration occurs [20–22]. Among female migrant workers in China, the longer the period of migration, the greater the hypertension risk [23]. However, one study also reported that migrants enjoyed significantly better physical health than rural residents but not urban dwellers [14]. The choice of the reference group for comparison clearly showed that migrants’ physical health outcomes were not necessarily poorer than their hometown counterparts.

Given the inconclusive results obtained for health and mental health outcomes between migrants and non-migrants, it is necessary to examine the nature of the association between migration and different types of health-related outcomes in a large-scale dataset. Various socioeconomic, psychosocial, and behavioral factors may mediate the effects of migration on health, including economic status, living standards, physical conditions, level of social support, information on and access to local health services, health-related investment, and lifestyle, etc. [10]. Although the existing studies have examined how the experience of discrimination influences health among migrants [5, 24], they pay less attention to whether migration makes individuals have more exposure to discrimination and then further influences their health. The “health depletion effect” proposes that migrants’ health deteriorates the longer they stay in the migration destination such that any health advantage migrants enjoy in the early stage of migration tends to diminish over time [3, 4]. A literature review have summarized that it is important to examine the impact of social, economic, environmental, emotional, and behavioral risk factors on migrants’ health in future research [16]. From the perspective of the health depletion effect, compared with other factors, discrimination experience could be one of the covariates that contribute to the negative association between migration and health. Yet, the nature of such an association is unclear. Discrimination experience often defined by how rural-to-urban migrants perceive unfair encounters and how they understand their experience in the context of social inequality and rural-urban disparities. And the structural factors, such as the household registration (*hukou* in Chinese) system and the conflict between individuals and governmental agencies, may help to elucidate the health depletion effect of migration in China.

### Migration and discrimination

Rural-to-urban migration in China is closely related to the country’s *hukou* system. The two main *hukou* types are agricultural and non-agricultural *hukou*. The system is highly selective, and it is very difficult to transfer one’s *hukou* status from agricultural *hukou* to non-agricultural *hukou* [8, 25]. Urban residents enjoy better public welfare and more social services, such as the public provision of schooling, healthcare, housing, and retirement benefits, relative to rural residents [25, 26]. In addition to *hukou* type, *hukou* location (*hukou suozaidi* in Chinese) also matters. The provision of public welfare and social services is based on *hukou* location, and local governments tend to lack incentives to provide services for migrants given that fiscal decentralization has made room for the exercise of a discretionary power [27, 28]. Rural-to-urban migrants hold agricultural, non-local *hukou*, which means they have restricted access to public welfare and social services in urban China. There has been recent *hukou* reform aimed at establishing a unified residential *hukou* without specifying the agricultural or non-agricultural type [29], but the welfare and social service reforms have not kept pace with the *hukou* reform. Accordingly, rural-to-urban migrants still do not have equal access to public welfare and social services. Indeed, such migrants are frequently treated as second-class citizens, occupy marginalized positions, and face a variety of institutional, economic, cultural, and social barriers in urban China [8, 9]. Apart from *hukou*-related discrimination, people have also faced other social discrimination. Based on the data from the China Labor Dynamics Survey (2012), one study revealed that current migrants were the least likely to perceive the fairness of current living standards compared with their efforts, while rural non-migrants were most likely to perceive the fairness of current living standard [30]. The types of discrimination migrants face including institutional discrimination, such as inequality of access to welfare and services, and interpersonal discrimination, which is observed during social encounters and interactions at the individual level [5, 9, 31]. The concept of self-perceptions of discrimination is often used in previous studies. Considering people may have differences in perceiving a given situation as discrimination, the perceived discrimination may be different from actual discrimination [32]. Nevertheless, self-perceived discrimination is found negatively associated with health and well-being, though the perception of discrimination is not verified using actual events [5, 33]. Perceiving discrimination also reflects people’s cognitive appraisal of a given situation, the self-report experiences can be stressful, and thus it is worthwhile to examine the effects of perceived discrimination [33, 34]. Given the possible discrimination faced by rural-to-urban migrants, one aim of the current study was to ascertain the effect of their perceived discrimination experience on the relationship between migration and health outcomes.

### Discrimination and health status

Discrimination experience has been identified as a risk factor for poor health among minorities and immigrants in the existing literature [33, 35]. However, these studies have employed different measures of discrimination and health. The experience of discrimination is negatively associated with self-reported health. For example, a study using data on 7720 valid responses to the Longitudinal Survey of Immigrants to Canada revealed visible minorities and immigrants who had experienced discrimination or unfair treatment to be the most likely to experience a decline in self-reported health status in Canada [36]. Experience of discrimination is also negatively associated with mental health and physical health. A literature review reported such experience to be associated with poor mental health outcomes among Latinos in the United States, with the studies reviewed employing measures of perceived discrimination, employment discrimination, and phenotype discrimination [37]. Unfair treatment was harmful to cardiovascular health, with cumulative unfair treatment associated with worse subclinical cardiovascular disease among Caucasian women [38]. A study reviewing 62 empirical articles that had used different measures of discrimination experience found such experience to be associated with poorer health among Asian Americans [35]. Most of the articles reviewed focused on mental health problems, although some examined the role of discrimination in physical health measures such as physical functioning, cardiovascular disease, and chronic physical condition. A meta-analytic review analyzed articles covering multiple forms of perceived discrimination and both mental and physical health outcomes and reported that perceived discrimination negatively influences both mental and physical health [33]. Some scholars have also differentiated between directly targeted discrimination (i.e., that experienced personally) and ambient forms thereof (i.e., witnessing, overhearing, or being aware of others’ discrimination experience), with both forms associated with negative psychosocial outcomes [39].

The foregoing studies were all conducted among minorities or immigrants in the international context, while studies carried out in China have also revealed exposure to discrimination or unfair treatment to be associated with poor health among rural-to-urban migrants. Rural-to-urban migrants are confronted with a marginalized life in urban China and, as a result, have poorer psychological health [9]. Employing data from a national household survey in 2009, it was found that perceived interpersonal discrimination has a more detrimental effect than perceived institutional discrimination on self-rated physical health and depressive distress among rural-to-urban migrants [5]. Chen’s measures of interpersonal and institutional discrimination focused on six types of behaviors and events, and the study left other types of discrimination unexamined and discrimination attributions unmentioned. A cross-sectional survey of 1006 rural-to-urban migrants in Beijing also revealed experienced or perceived unfair treatment, including job and workplace discrimination, distrust, negative attitudes among others, and unfair treatment from local law enforcement, to be negatively linked to the quality of life, including overall well-being, general health, physical health, psychological health, social relationships, and environment scores [6]. Another analysis of cross-sectional data in Beijing also revealed that both experiences of discrimination and perceived social inequity were strongly related to the mental health problems of rural-to-urban migrants [24]. However, the study sample was restricted to rural-to-urban migrants in Beijing, and the relationship between discrimination and quality of life and mental health in these two studies was examined without a reference group. In summary, although previous studies have adopted different measures of unfair treatment and discrimination, exposure to unfair treatment and discrimination is generally associated with poorer health, both in China and internationally.

### Factors influencing migration decisions

Migrants tend to be a self-selected group among those who determine to migrate. Thus, migration selection is not random, and factors, such as the pre-migration statuses of health and mental health that may support a decision of migration are critical to be adjusted when testing the health consequences of migration [12]. Variables used in previous studies to predict migration decisions are included in this study, and these variables include gender, age, age squared, ethnic minority, education level, logarithm of household income, household size, and regions [12, 40, 41]. Health is identified to be an important factor influencing migration decisions: individuals who report better health are more likely to migrate [42, 43], and those who have poorer health are more likely to return to their hometown [42, 44]. In addition, compared with the first-generation migrants, the new generation migrants are more willing and adaptable to stay in the city [45], and thus cohort is also included as a determinant of migration. Considering different factors may influence migration decisions and further affect health status after migration, particular methods are required to reduce the selection bias before analyzing the relationship between migration and health.

### Research gaps and hypotheses

The existing literature has revealed inconsistent health differences between migrants and non-migrants and identified the complicated influence of migration on health. It is important to examine the relevant pathways on how migration exerts influence on health, and the current study hypothesizes that migration poses a negative impact on health and mental health in consideration of discrimination experience. As informed by the literature review, the indirect effect of discrimination experience on the relationship between migration and health status is underexplored. Besides, migrants are a self-selected group. In order to rule out the possible selection bias before examining the health consequences of migration, a rigorous data analytical method, such as the propensity scoring method is adopted.

**Fig 1:**
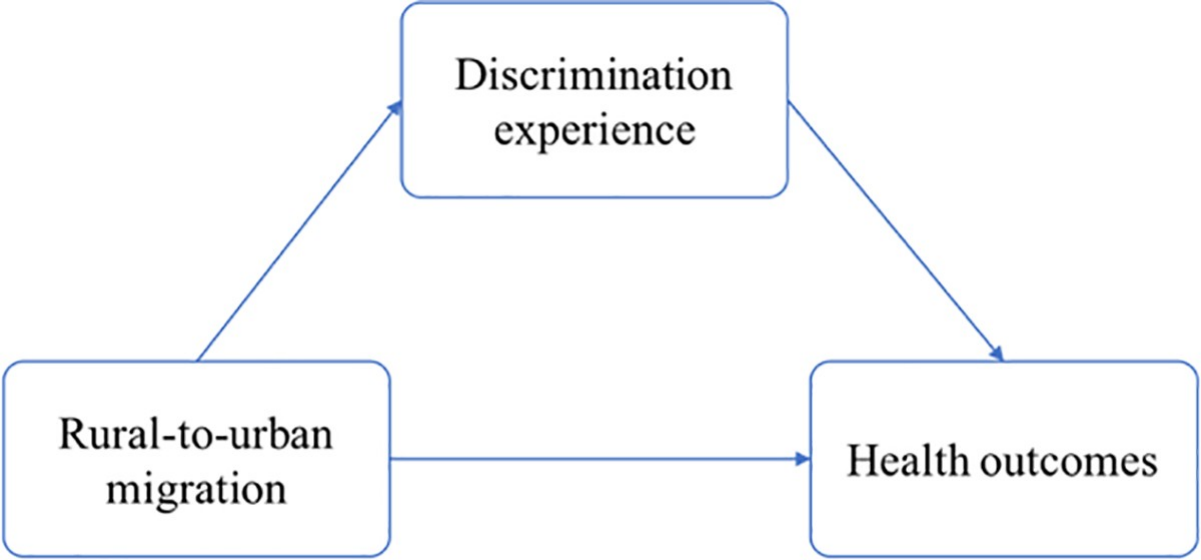
Framework of rural-to-urban migration, discrimination experience, and health outcomes.

This study was designed to answer the following research question: Does discrimination experience mediate the associations between migration status and health outcomes, such as self-reported health, mental health, and physical health? The study has three hypotheses: (1) Discrimination experience mediates the relationship between rural-to-urban migration and self-reported health. (2) Discrimination experience mediates the relationship between rural-to-urban migration and mental health. (3) Discrimination experience mediates the relationship between rural-to-urban migration and physical health. Fig 1 depicts the hypothesized relationship among rural-to-urban migration, discrimination experience, and health outcomes.

## Methods

### Data

An ethics approval of the research was granted by the Human Research Ethics Committee (HREC), The University of Hong Kong (Reference No. EA1708010). China Family Panel Studies (CFPS), launched by the Peking University Institute of Social Science Survey (ISSS) in 2010, provide high-quality, nationally representative, longitudinal data [46]. The CFPS data can be obtained from this website (https://opendata.pku.edu.cn). The topics covered by the CFPS surveys include economic status, education, family dynamics and relationships, migration, and health, among others. The baseline survey was conducted in 2010, with separate follow-up surveys conducted in 2012, 2014, 2016, and 2018. The 2010 baseline survey adopted multi-stage (i.e., county, village, and household) probability-proportional-to-size sampling with implicit stratification. All members of sampled households aged 9 or above were interviewed, with the total sample comprising 14,960 households and 33,600 adults from 635 communities across 25 provinces, municipalities, and autonomous regions [47].

Using data from the cross-sectional component of the third (2014) CFPS wave, this study examined whether discrimination experience mediates the relationship between migration and health. Although CFPS 2014 does not provide the latest data, its measure of discrimination experience includes three answers (i.e., “neither heard nor experienced”, “have heard about it, but haven’t experienced”, and “have experienced”), whereas the measure in CFPS 2016 includes only two (i.e., have experienced and have not experienced), and CFPS 2018 does not ask about respondents’ discrimination experience. Hearing about discrimination in CFPS 2014 captures the experience of ambient forms of discrimination, and is associated with awareness of stigmatized status, anxiety, and depression [39, 48]. Thus, data from CFPS 2014 were used to conduct the analysis herein. Compared with the 2010 sample, the response rate to the second follow-up survey in 2014 was 79.3% [49], which is quite a high response rate. The analytical sample is limited to those who were within working-age and still participated in the labor market. Respondents who were out of the labor market and who were younger than 16 or older than 60 were excluded.

### Analytical strategy

Stata 16 was employed to conduct the analysis. Descriptive analysis, chi-square tests, and t-tests were first performed to show the general features of rural residents and rural-to-urban migrants. Then, to resolve rural-to-urban migration selection bias, the PSM method was adopted, with propensity scores computed via logistic regression and 1:1 nearest neighbor matching conducted with a caliper of 0.25*standard deviation (SD). Stata with “psmatch2” was used for PSM [50]. Samples whose propensity scores were beyond the common support were excluded in the matching procedures. According to the previous research, this matching technique can match each rural-to-urban migrant with a rural resident with observable characteristics such that the probability of being a rural-to-urban migrant is very similar for the migrant and non-migrant [51]. Subsequently, the average treatment effect of migration on discrimination experience and health can be obtained by comparing the matched rural-to-urban migrants and rural residents in terms of their discrimination experience and health status.

Finally, regression and mediation analyses were conducted after PSM. For regression analysis, logistic regression was performed for self-reported health and physical discomfort, whereas ordinary least squares (OLS) regression was performed for psychological distress and discrimination experience. For mediation analysis, the natural indirect effects (NIE) were computed through the “paramed” program in Stata [52, 53]. The NIE, formally defined as NIE = E[Y_1_M_1_-Y_1_M_0_], compares the counterfactual outcome of the mediator value of X = 1 and X = 0, with the treatment status fixed as X = 1 [54, 55]. Specifically, NIE in this research answers this counterfactual question: If we were to hold migration status as rural-to-urban migration, what would be the effect on the health status of a change in the level of discrimination experience from the value realized for rural residents to the value realized for rural-to-urban migrants? It evaluates how much the health outcomes would change on average if the rural-to-urban migration’s influence exerted only through modifying the discrimination experience [55]. The NIE helps to compare the change of contrasting the discrimination experience with the exposure fixed at the rural-to-urban migration status. Examining the NIE helps us to understand how rural-to-urban migration negatively influences health outcomes through discrimination experience from the perspective of health depletion effect, which has not been fully studied in previous research. Evaluating the NIE is policy relevance. If anti-discrimination practices and policies could be provided for the rural-to-urban migrants, their health outcomes may therefore be improved. Bootstrapping with 1000 replications was employed for the bias-corrected confidence interval (CI). The seed was set as 1234. Linear regressions were performed to fit discrimination experience and psychological distress, and logistic regressions for such dichotomized outcomes as self-reported health and physical discomfort. No treatment-mediator interaction was included in the models.

Sensitivity analysis and results comparison are important in propensity score analysis [56]. Different calipers were employed to perform the matching for the estimated propensity score. The first caliper was set at the recommended size of a quarter of an SD of the estimated propensity score, whereas the narrowest caliper was set at 0.05 and the widest at 0.1 and 0.5 [56].

Only respondents with no missing values for the mediator, independent, dependent, and control variables were selected for data analysis. Different sets of control variables were employed in the propensity score computation and mediation analysis, as discussed in the following section. For the chi-square tests, t-tests, and regression analysis, coefficients with p values smaller than 0.05 were considered significant. For mediation analysis using bootstrapping, indirect effects were considered to exist if the 95% bias-corrected CI for the odds ratio (OR) did not contain the value of 1 [57] or that for the coefficients using linear regressions did not include the value of 0.

### Variables

#### Independent variable

Similar to other migration studies using national data [5, 58], migration status was differentiated by respondents’ *hukou* status and residence during the survey period (in a rural or urban area) in this study. Although minimum migration time was not included in Chen’s and Wang’s definitions, being away from one’s permanent *hukou* residence for at least six months was required to define a rural-to-urban migrant in this study. Accordingly, rural residents are defined as individuals who hold an agricultural *hukou* and live in a rural area, whereas rural-to-urban migrants are individuals who hold an agricultural *hukou* but lived in an urban area for at least six months during the survey period. Individuals who remain in their rural locale were selected as a comparison group for rural-to-urban migrants to measure the health consequences of migration, as selecting those living in the urban destinations might not have allowed us to differentiate between migration’s effects on health and preexisting health disparities between the sending and receiving locales [10, 59].

#### Dependent variables

Three measures of health-related outcomes, namely, self-reported health, psychological distress, and physical discomfort, were included as dependent variables. Self-reported health was measured by a single item, “How would you rate your health status?”, with the answers re-coded as a dichotomized variable (see Table 1). The six-item screening scale for psychological distress [60] was adopted to assess mental health. This scale measures respondents’ frequency of feeling depressed, nervous, restless or fidgety, hopeless, and that everything was an effort or meaningless during the past month, with the answers re-coded as 1 = “Never,” 2 = “Sometimes,” 3 = “Half the time,” 4 = “Often,” and 5 = “Almost every day.” The composite score was calculated by summing all of the items, and higher values indicate the severity of psychological distress. The Cronbach’s alpha among all adult respondents in 2014 was .858 (N = 31418). Finally, physical discomfort was assessed by a single item: “During the past two weeks, have you felt any physical discomfort?” This item shows the respondents’ physical health. The coding values of answers were shown in Table 1.

**Table 1:** Study sample characteristics by migration status before PSM.

Mean (SD)/Frequency (%)	Total sample	Rural residents	Rural-to-urban migrants	*p*
N	8,854 (100.00%)	8,228 (92.93%)	626 (7.07%)	
Self-reported health				< 0.001^a^
Poor or fair health (= 0)	2,191 (24.75%)	2,093 (25.44%)	98 (15.65%)	
Good health (= 1)	6,663 (75.25%)	6,135 (74.56%)	528 (84.35%)	
Psychological distress during the past month	9.161 (3.816)	9.162 (3.826)	9.149 (3.680)	0.931^b^
Physical discomfort during past two weeks				0.009^a^
No (= 0)	6,482 (73.21%)	5,996 (72.87%)	486 (77.64%)	
Yes (= 1)	2,372 (26.79%)	2,232 (27.13%)	140 (22.36%)	
Self-reported change in health	.852 (.585)	.846 (.589)	.930 (.535)	< 0.001^b^
Discrimination experience	2.086 (2.968)	2.051 (2.959)	2.553 (3.048)	< 0.001^b^
Gender				0.052^a^
Male (= 0)	4,799 (54.20%)	4,483 (54.48%)	316 (50.48%)	
Female (= 1)	4,055 (45.80%)	3,745 (45.52%)	310 (49.52%)	
Age (in years, 16–60)	41.289 (11.138)	41.691 (11.147)	35.998 (9.560)	< 0.001^b^
Age squared	18.288 (9.031)	18.624 (9.059)	13.871 (7.347)	< 0.001^b^
Cohort				< 0.001^a^
New generation (= 0, born in or after 1980)	2,678 (30.25%)	2,368 (28.78%)	310 (49.52%)	
First-generation (= 1, born before1980)	6,176 (69.75%)	5,860 (71.22%)	316 (50.48%)	
Ethnicity				< 0.001^a^
Minority ethnicity (= 0)	882 (9.96%)	854 (10.38%)	28 (4.47%)	
Han ethnicity (= 1)	7,972 (90.04%)	7,374 (89.62%)	598 (95.53%)	
Marital status				0.948^a^
Never married (reference)	778 (8.79%)	723 (8.79%)	55 (8.79%)	
Divorced or widowed	264 (2.98%)	244 (2.97%)	20 (3.19%)	
Married or cohabiting	7,812 (88.23%)	7,261 (88.25%)	551 (88.02%)	
Employment status				< 0.001^a^
Unemployed (= 0)	95 (1.07%)	78 (0.95%)	17 (2.72%)	
Employed (= 1)	8,759 (98.93%)	8,150 (99.05%)	609 (97.28%)	
Education level				< 0.001^a^
Schooled for up to 6 years (reference)	4,687 (52.94%)	4,481 (54.46%)	206 (32.91%)	
Schooled for 6–12 years	3,866 (43.66%)	3,527 (42.87%)	339 (54.15%)	
More than 12 years of schooling	301 (3.40%)	220 (2.67%)	81 (12.94%)	
Medical insurance				< 0.001^a^
Without medical insurance (= 0)	540 (6.10%)	453 (5.51%)	87 (13.90%)	
With medical insurance (= 1)	8,314 (93.90%)	7,775 (94.49%)	539 (86.10%)	
Religious belief				0.239^a^
No religous belief (= 0)	6,541 (73.88%)	6,091 (74.03%)	450 (71.88%)	
Have religious belief (= 1)	2,313 (26.12%)	2,137 (25.97%)	176 (28.12%)	
Net family income per capita (ln)	8.929 (1.179)	8.876 (1.180)	9.629 (.909)	< 0.001^b^
Household size	4.639 (1.944)	4.707 (1.950)	3.749 (1.609)	< 0.001^b^
Regions				< 0.001^a^
Eastern Region (reference)	2,466 (27.85%)	2,168 (26.35%)	298 (47.60%)	
Central Region	2,254 (25.46%)	2,133 (25.92%)	121 (19.33%)	
Western Region	2,983 (33.69%)	2,901 (35.26%)	82 (13.10%)	
Northeast Region	1,151 (13.00%)	1,026 (12.47%)	125 (19.97%)	

Note

^a^ p values are from the chi-square tests

^b^ p values are from the t-tests (two-tailed).

#### Mediator

Discrimination experience was measured by the experience of life events perceived to be unfair and attributed to a specific cause such as inequality or *hukou* status. CFPS 2014 includes seven self-reported items concerning discrimination experienced in the past year: “Unfair treatment due to inequality between the rich and the poor”; “Unfair treatment due to household registration status”; “Unfair treatment due to gender discrimination”; “Unfair treatment by government officials”; “Conflict with government officials”; “Unreasonable delay and stalling at a government agency”; and “Unreasonable charges paid to a government agency.” The answers for each item were recoded as 0 = “Neither heard nor experienced,” 1 = “Have heard about it, but haven’t experienced,” and 2 = “Have experienced.” The composite score of the seven items was considered as a continuous variable, with an acceptable Cronbach’s alpha of .800 (N = 30105) among all adult respondents in 2014. Higher values indicate the severity of discrimination experience.

#### Control variables

Basic demographic and socioeconomic variables were included as control variables, including gender, age, age squared, cohort, ethnicity, marital status, employment, education level, religious belief, medical insurance, logged per capita annual household income, household size, and regional information. These variables are commonly used in previous studies focusing on the health status of migrants in China. Table 1 shows the coding values of gender, cohort, ethnicity, employment status, medical insurance, and religious belief, and the recategorized dummy variables of marital status and education level. Household income was adjusted by household size, i.e., net family income per capita (yuan) and naturally log-transformed. The value of “1” was added to the family income of all respondents to use the log function. Household size refers to the number of family members who live in the household or who do not live at home but have close financial ties to other members such as many migrants. The household income and household size data were complemented with the family-level datasets. Four dummy variables, namely, “Eastern Region,” “Central Region,” “Western Region,” and “Northeast Region,” were employed to represent the regions in which the respondents were surveyed. The “paramed” command uses the same set of covariates to estimate two models: one is for the mediator conditional on treatment and covariates, and the other one is for the outcome conditional on treatment, the mediator, and covariates [53].

A different set of variables was used to estimate the propensity scores, including gender, age, age squared, cohort, ethnicity, marital status, employment status, education level, natural log of net family income per capita, household size, and regional information, and self-reported change in health. Self-reported change in health was assessed by respondents’ rating of their health status during the survey period compared to the previous year (0 = “Worse,” 1 = “No change,” and 2 = “Better”).

## Results

### Descriptive statistics, chi-square tests, and t-tests before PSM

Descriptive analysis was first conducted to ascertain the differences between rural-to-urban migrants and rural residents. Table 1 depicts the sample characteristics of migration statuses. It can be seen that rural residents accounted for 92.93% of the sample (N = 8,228), with just 7.07% rural-to-urban migrants (N = 626). The migrants had higher proportions of good self-reported health (84.35%), and lower proportions of physical discomfort in the past two weeks (22.36%) than rural residents, but there was no significant difference in psychological distress between the two groups (rural residents: Mean [M] = 9.162 [SD = 3.826] versus rural-to-urban migrants: M = 9.149 [SD = 3.680]; *p* > 0.05). However, the rural-to-urban migrants reported higher discrimination experience scores (M = 2.553 [SD = 3.048]) than the rural residents (M = 2.051 [SD = 2.959]). There were significant differences between the two groups for the majority of control variables except for gender, marital status, and religious belief. Detailed results are shown in Table 1.

### Results of propensity score estimation

A propensity score was estimated via logistic regression using a set of variables that might explain why some people are more likely than others to become rural-to-urban migrants. Results of propensity score estimation show that individuals who were female, in the middle age, belonged to the Han ethnicity, were divorced or widowed or married or cohabiting, had received 6–12 or 12+ years of schooling, and/or had a higher net family income per capita were more likely to be a rural-to-urban migrant than a rural resident. In contrast, individuals who were younger or older, employed, living in a large household, and/or living in the Central Region or Western Region were less likely to be a rural-to-urban migrant. The detailed results are presented in S1 Table in the supporting information.

Samples whose propensity scores were beyond the common support were excluded from the matching procedures. The common support of treated and untreated samples, the histograms of the estimated propensity scores by treatment status, and the standardized % bias across covariates after PSM declined for all of the matched samples are provided in S1–S3 Figs, respectively, in the supporting information. The post-PSM chi-square tests and t-tests also revealed no significant differences between rural residents and rural-to-urban migrants for any of the aforementioned variables used to ascertain the determinants of rural-to-urban migrant status. The Rubin’s B for the matched sample was 12.9, which is smaller than 25 and thus acceptable; the Rubin’s R was 0.96, which is within the acceptable level of .5 to 2 [61, 62]. The combination of balancing tests found the balancing property to be generally satisfied.

### Results of regression and mediation analyses after PSM

The new sample generated by PSM (rural residents: N = 609; rural-to-urban migrants: N = 609) was employed to conduct mediation analysis. Table 2 reports the associations between rural-to-urban migration and discrimination experience and the three measures of health. Rural-to-urban migration was positively linked to perceived discrimination experience (β = 0.768, *p* < 0.001), which indicates that rural-to-urban migrants reported more discrimination experience than rural residents. The associations between migration and self-reported health (OR = 1.069, *p* > 0.05) and physical discomfort (OR = 1.169, *p* > 0.05) were not significant, whereas migration was positively associated with psychological distress (β = 0.516, *p* < 0.01).

**Table 2:** Results of regression analysis after PSM.

	(1)	(2)	(3)	(4)
	Discrimination experience	Self-reported health ^a^	Psychological distress	Physical discomfort ^a^
Migration	0.768***	1.069	0.516**	1.169
	(0.163)	(0.176)	(0.194)	(0.172)
Gender	-0.429**	0.668*	0.585**	2.423***
	(0.164)	(0.111)	(0.196)	(0.368)
Age	0.089	0.957	0.067	0.995
	(0.085)	(0.092)	(0.102)	(0.076)
Age squared	-0.100	0.990	-0.059	1.047
	(0.100)	(0.106)	(0.119)	(0.092)
Cohort	-0.325	1.161	-0.570	0.762
	(0.340)	(0.427)	(0.405)	(0.238)
Ethnicity	-0.723^!^	0.950	-0.086	0.755
	(0.406)	(0.379)	(0.483)	(0.255)
Divorced or widowed	-0.228	0.306^!^	1.371*	1.782
	(0.583)	(0.216)	(0.694)	(0.883)
Married or cohabiting	-0.108	0.356^!^	0.071	0.993
	(0.346)	(0.201)	(0.413)	(0.324)
Employment	-0.281	1.158	0.092	0.937
	(0.510)	(0.619)	(0.608)	(0.413)
Schooled for 6–12 years	0.185	1.521*	0.158	0.684*
	(0.183)	(0.264)	(0.218)	(0.109)
More than 12 years of schooling	0.175	2.060^!^	0.172	0.711
	(0.306)	(0.803)	(0.364)	(0.199)
Medical insurance	0.438	0.907	-0.748*	1.010
	(0.272)	(0.268)	(0.324)	(0.247)
Religious belief	0.490**	0.536***	0.601**	1.396*
	(0.187)	(0.093)	(0.223)	(0.225)
Net family income per capita (ln)	-0.089	0.945	-0.254*	0.993
	(0.098)	(0.092)	(0.117)	(0.085)
Household size	0.012	1.082	-0.093	0.873**
	(0.056)	(0.063)	(0.067)	(0.045)
Central Region	-0.147	1.041	0.359	1.497*
	(0.225)	(0.240)	(0.268)	(0.297)
Western Region	0.923***	0.772	1.064***	1.487^!^
	(0.263)	(0.195)	(0.314)	(0.341)
Northeast Region	-0.273	1.373	-0.011	1.049
	(0.219)	(0.316)	(0.261)	(0.210)
Constant	1.536	105.927*	9.852***	0.266
	(1.887)	(226.818)	(2.247)	(0.451)
*N*	1218	1218	1218	1218
*R*^2^	0.055		0.047	
adj. *R*^2^	0.040		0.033	
pseudo *R*^2^		0.082		0.060

Note

^a^ Exponentiated coefficients; Standard errors in parentheses.

! *p* < 0.1

* *p* < 0.05

** *p* < 0.01

*** *p* < 0.001.

Table 3 presents the results of post-PSM mediation analysis. In general, the indirect effects of discrimination experience on the relationships between rural-to-urban migration and the three measures of health were significant in this analysis with controlling for relevant variables. More specifically, holding migration status as rural-to-urban migration, for a change in the level of discrimination experienced from the value realized for rural residents to the value realized for rural-to-urban migrants, the OR of good self-reported health was 0.926 (95% CI: 0.868 0.969), indicating lower odds of good self-reported health for the migrants. Again, holding migration status as rural-to-urban migration, for a change in the level of discrimination experienced from the value realized for rural residents to the value realized for rural-to-urban migrants, the value of psychological distress increased by 0.258 (95% CI: 0.152 0.409), and for a change in the level of discrimination experienced from the value realized for rural residents to the value realized for rural-to-urban migrants, the OR of physical discomfort was 1.096 (95% CI: 1.042 1.171), indicating higher odds of reporting physical discomfort. In addition, the total effect of rural-to-urban migration on psychological distress was significant, and rural-to-urban migration lead to psychological distress increasing by 0.516 (95% CI: 0.114 0.895). Other effects were not significant.

**Table 3:** Results of mediation analysis after PSM.

N = 1218		Self-reported health ^a^^,^^b^	Psychological distress	Physical discomfort ^a^
Controlled direct effect	coef	1.168	0.258	1.068
	BC	(0.857 1.666)	(-0.112 0.627)	(0.771 1.436)
Natural indirect effect	coef	**0.926**	**0.258**	**1.096**
	BC	(0.868 0.969)	(0.152 0.409)	(1.042 1.171)
Total effect	coef	1.082	**0.516**	1.170
	BC	(0.801 1.552)	(0.114 0.895)	(0.842 1.565)

Note: BC = bias-corrected confidence interval; Coefficients in bold are significant.

^a^ Odds ratio.

^b^ One or more parameters could not be estimated in 9 bootstrap replicates; standard-error estimates include only complete replications.

### Sensitivity analysis

The results of sensitivity analysis using different calipers in PSM for regression analysis and mediation analysis are presented in S2 and S3 Tables, respectively, in the supporting information. In general, sensitivity analysis after PSM using different calipers yielded similar results to that in which the caliper was set at 0.25*SD, thereby indicating the robustness of the results. For the regression analysis after PSM using different calipers, rural-to-urban migration remained positively linked to discrimination experience and psychological distress, whereas post-PSM such mediation analysis revealed discrimination experience to have significant indirect effects on the relationship between migration and three measures of health. The total effects of migration on psychological distress were also statistically significant.

## Discussion

Drawing on data from CFPS 2014, the study reported herein explored how rural-to-urban migration changes individuals’ discrimination experience and to what extent such experience mediates the relationship between rural-to-urban migration and three measures of health. Previous research has documented the negative health impacts of discrimination experience, but the issue of how discrimination experience mediates the relationship between rural-to-urban migration and health outcomes, including self-reported health, psychological distress, and physical discomfort, has been relatively neglected. The current research considered both ambient forms of discrimination and directly targeted discrimination in its measures of discrimination experience and provides the evidence needed to explore the indirect effect of such experience.

Few if any studies in the extant literature have resolved the possible self-selection bias in rural-to-urban migration status before examining the potential relationship between migration and health. In this research, we estimated propensity scores to match each rural-to-urban migrant in our sample with a rural resident who had a similar probability of migration, thereby resolving the potential self-selection bias in such research [12]. Rural-to-urban migrants are a self-selected group, and factors such as health may influence individuals’ migration decision and further influence their discrimination experience and health outcomes after migration. For instance, if persons who have higher levels of education are more likely to migrate, and education level is related to health, then the health disparity between the migrants and non-migrants may not be the result of migration but it is mixed with factors such as education level. Thus, migrants’ self-selection bias is considered through conducting propensity score matching in this study. In addition, we also conducted a sensitivity analysis by adopting different calipers in the matching procedures, thereby helping us to compare our results and determine whether they are robust.

Post-PSM regression analysis demonstrated rural-to-urban migration to be positively associated with discrimination experience, with rural-to-urban migrants reporting more discrimination after migrating to urban areas. This finding is inconsistent with Chen’s study showing that migrants do not perceive more discrimination than non-migrants with similar sociodemographic characteristics [5]. One possible explanation is that the two studies adopted different measures of discrimination, and Chen also did not control for potential selection bias before comparing the discrimination experience of migrants and non-migrants. It is understandable that rural-to-urban migrants would report more discrimination experience than rural residents, as the latter have fewer opportunities to communicate with urban residents and urban government agencies, and thus a more localized frame of reference concerning their circumstances [30].

Post-PSM mediation analysis revealed migration to exert a significant total effect on psychological distress, which indicates that rural-to-urban migration is related to higher values of psychological distress. However, the migration’s total effects on self-reported health and physical discomfort were not statistically significant. Psychological distress measures people’s mental status during the past month, while self-reported health is a more general and longer-term measure of health, and physical discomfort focuses exclusively on physical aspects of health in the past two weeks. The results suggest that experience of discrimination may have a more harmful effect on people’s mental health rather than self-reported health and physical health. This is consistent with previous research that perceived discrimination is more strongly associated with mental health than physical health [63]. This research also identified significant indirect effects of discrimination experience on the relationship between migration and the three measures of health. Holding migration status as rural-to-urban migration, more experience of discrimination is linked to poorer health, including lower odds of self-reporting good health, higher scores of psychological distress, and higher odds of reporting physical discomfort. Although the total effects of rural-to-urban migration on self-reported health and physical discomfort are not significant, it is still necessary to examine the indirect effects of migration on health. Recent research on mediation analysis also recommends that a significant test for total effects should not be employed as a prerequisite for a test of indirect effects in studies focusing on mediation alone [64]. Discrimination experience reflects how rural-to-urban migrants perceive unfair encounters in the context of social inequality and rural-urban disparities in China, thereby adding to our understanding of the health depletion effect of migration.

After rural individuals migrate to urban areas, they have more exposure to discrimination than previously, and those experiencing more discrimination often have worse self-reported health, more psychological distress, and more physical discomfort. Appropriate practices and policies are thus required to deal with unfair treatment and discrimination during the process of rural-to-urban migration. The discrimination experience measured in the current research pertained to inequality between the rich and the poor, *hukou* status- and gender-based discrimination, and discrimination by government officials and government agencies. Thus, it is important to promote social equality, gender equality, and social inclusion. The reform of the *hukou* system and better public welfare and social service arrangements are also needed. Although the Chinese government has reformed the *hukou* system to establish a unified residential *hukou* without agricultural and non-agricultural types, welfare and social service reforms have not kept pace, meaning that rural-to-urban migrants still face a variety of institutional barriers and do not have equal access to welfare. Further *hukou* reform is required to grant migrants easier and fairer access to public welfare and social services and to ensure equity in the provision of healthcare and public education to migrants and non-migrants [28, 65, 66]. In addition, government officials should be supervised to ensure that they provide a fair and reasonable service, and the work of government agencies should be evaluated in accordance with the rules of openness and transparency. It should also be noted that this study not only differentiated between “have experienced discrimination” and a lack thereof, but also considered ambient forms of discrimination linked to awareness of stigmatized status, anxiety, and depression [39, 48]. Living in an environment in which one hears about or witnesses others being treated unfairly may make one feel anxious about his or her own disadvantaged position [39, 48]. Building a migrant-friendly environment is thus of the utmost importance.

Although it makes important contributions to the literature, this research had several limitations that must be acknowledged. First, rural residents may include both rural local residents and rural-to-rural migrants, and thus both were included in our group for comparison with rural-to-urban migrants. Second, the data used in this research were cross-sectional in nature, and thus only cross-individual differences in discrimination experience and health were examined. Longitudinal mediation analysis is required to test how discrimination experience mediates the relationship between migration and health over the long term, with both cross-individual differences and individual change over time considered. Third, this research explored only whether the frequency of discrimination experience plays a role in the relationship between migration and health, with the indirect effects of specific types of discrimination experience on that relationship left unexamined. The way in which different types of discrimination experience influence the relationship between migration and health would be a fruitful direction for future research. In addition, there may be other types of discrimination which are not included in the seven items. Including more types of discrimination experience would be helpful to examine the effects of discrimination. Fourth, the three outcome variables, namely, SRH, psychological distress, and physical discomfort, were included in three independent models due to the limitation of using “paramed” that multiple outcomes cannot be included simultaneously. Considering that the three outcome variables are associated with each other, employing them as mutually independent variables may result in an inflation of Type I error. Fifth, as the CFPS adopts multi-stage probability sampling with implicit stratification, survey design effects (stratum, cluster, and individual weight) should have been included in estimating the parameters, but, technically, were not applied in our analysis. As two of the dependent variables, namely, self-reported health and physical discomfort, were dummy variables, “paramed” was adopted for mediation analysis, but does not support survey-weighted analysis [67, 68]. Given that it did not consider weight, stratification, or clustering, this research was unable to take full advantage of the CFPS’s nationally representative sample, and the estimated parameters may also have reflected a certain degree of bias [69]. Sixth, although the method of propensity score matching has addressed the selection-bias for the migration status, the selection-bias for the mediator, i.e., discrimination experience, probably remained unresolved [70, 71]. It is noted that blending a non-randomized binary treatment (migration status) and a non-randomized continuous mediator (discrimination experience) in the model may still subject to risk of self-selection bias. However, this research controlled for variables influencing both migration status and discrimination experience, which blocked the back-door path from migration status to health outcomes [72]. Nevertheless, although this research has controlled for many relevant variables, there may still be variables only influencing the mediator and not influencing the treatment, and not controlling for such variables may lead to a decrease of precision of the results to a certain extent [72]. Finally, this research was quantitative in nature. Obtaining qualitative data from rural-to-urban migrants and rural non-migrants would be conducive to a better understanding of the complicated associations among migration, experience of unfair treatment, and health.

## Conclusions

From the perspective of the health depletion effect, this research examines whether the experience of discrimination mediates the relationship between rural-to-urban migration and three measures of health in consideration of migration selection bias. After migrating from rural to urban areas, individuals have more exposure to discrimination, which is harmful to their self-reported health, mental health, and physical health. Specifically, rural-to-urban migration is positively associated with psychological distress, and policies and practices, such as reform of welfare and services and building a migrant-friendly society, are needed to help rural-to-urban migrants cope with psychological distress during the process of migration. This research provides empirical evidence for reducing social and institutional discrimination against rural-to-urban migrants and ensuring them benefit from the social and economic development in China.

## Supporting information

S1 Fig Common support of treated and untreated sample. (DOCX)

S2 Fig Histograms of estimated propensity scores by treatment status. (DOCX)

S3 Fig Standardized % bias across covariates after PSM. (DOCX)

S1 Table Logistic regression predicting propensity score of being a rural-to-urban migrant. (DOCX)

S2 Table Results of sensitivity analysis via regressions using different calipers in PSM. (DOCX)

S3 Table Results of sensitivity analysis by mediation analysis using different calipers in PSM. (DOCX)

S1 File (DTA)
