# Supplementary figures and images for: Rural-to-urban migration, discrimination experience, and health in China: Evidence from propensity score analysis
Source: PLoS One. 2020 Dec 28;15(12):e0244441. doi: 10.1371/journal.pone.0244441 (PMC7769422; doi:10.1371/journal.pone.0244441)

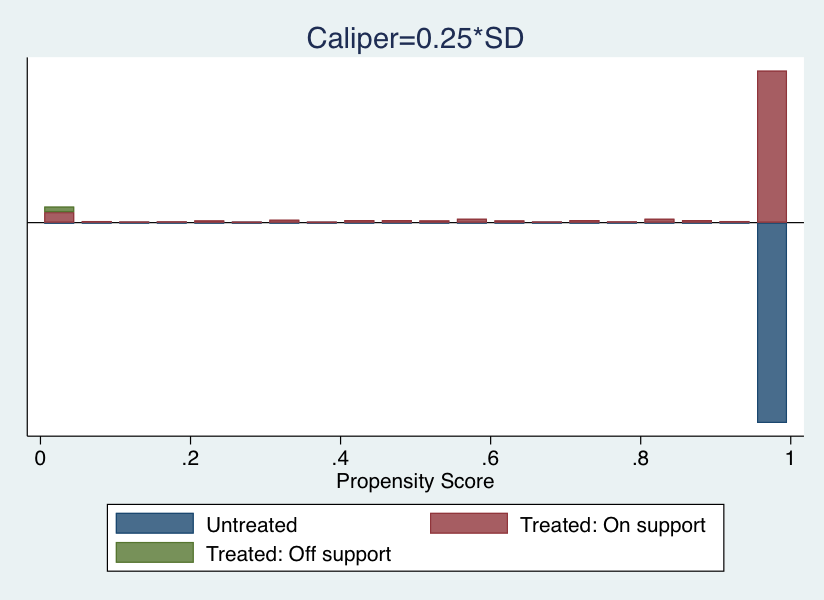


S1 Fig. Common support of treated and untreated sample

Supplement: S1 Fig — (DOCX) [file pone.0244441.s001.docx]

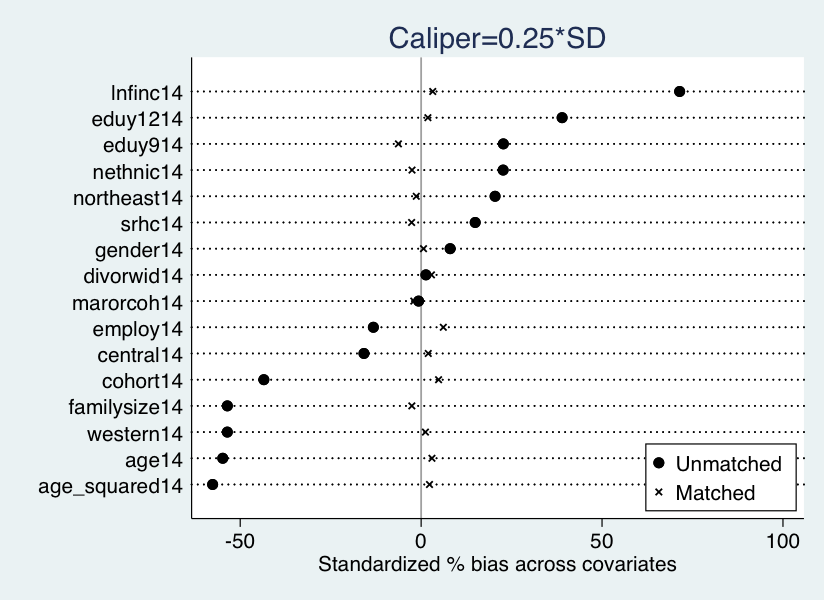


S3 Fig. Standardized % bias across covariates after PSM

Supplement: S3 Fig — (DOCX) [file pone.0244441.s003.docx]
