# Supplementary material for: Rural-to-urban migration, discrimination experience, and health in China: Evidence from propensity score analysis
Source: PLoS One. 2020 Dec 28;15(12):e0244441. doi: 10.1371/journal.pone.0244441 (PMC7769422; doi:10.1371/journal.pone.0244441)
Supplement: S2 Fig — (DOCX) [file pone.0244441.s002.docx]

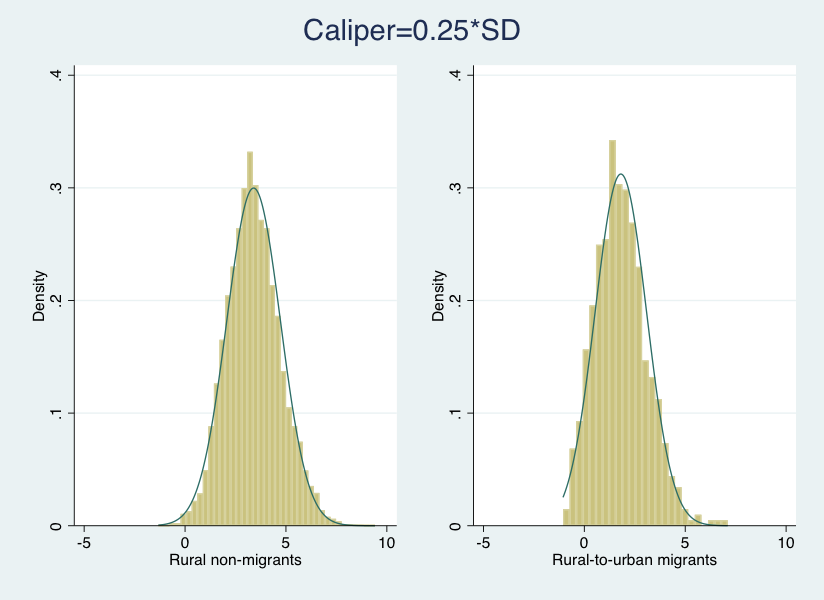


S2 Fig. Histograms of estimated propensity scores by treatment status

Note: The term of “Rural non-migrants” in this figure means “rural residents”.
