# Supplementary material for: Rural-to-urban migration, discrimination experience, and health in China: Evidence from propensity score analysis
Source: PLoS One. 2020 Dec 28;15(12):e0244441. doi: 10.1371/journal.pone.0244441 (PMC7769422; doi:10.1371/journal.pone.0244441)
Supplement: S1 Table — (DOCX) [file pone.0244441.s004.docx]

S1 Table. Logistic regression predicting propensity score of being a rural-to-urban migrant

|  | Migration status |
| --- | --- |
| Self-reported change in health | 1.102 |
|  | (0.093) |
| Gender | 1.215^*^ |
|  | (0.110) |
| Age | 1.217^***^ |
|  | (0.058) |
| Age squared | 0.731^***^ |
|  | (0.041) |
| Cohort | 0.767 |
|  | (0.148) |
| Ethnicity | 1.727^**^ |
|  | (0.365) |
| Divorced or widowed | 3.015^***^ |
|  | (0.945) |
| Married or cohabiting | 3.024^***^ |
|  | (0.566) |
| Employment | 0.412^**^ |
|  | (0.124) |
| Schooled for 6-12 years | 1.559^***^ |
|  | (0.155) |
| More than 12 years of schooling | 3.898^***^ |
|  | (0.683) |
| Net family income per capita (ln) | 1.568^***^ |
|  | (0.083) |
| Household size | 0.743^***^ |
|  | (0.023) |
| Central Region | 0.491^***^ |
|  | (0.059) |
| Western Region | 0.326^***^ |
|  | (0.044) |
| Northeast Region | 0.898 |
|  | (0.110) |
| Constant | 0.000^***^ |
|  | (0.000) |
| *N* | 8854 |
| pseudo *R*^2^ | 0.186 |

Note: Exponentiated coefficients; Standard errors in parentheses

! *p* < 0.1, * *p* < 0.05, ** *p* < 0.01, *** *p* < 0.001
