# Supplementary material for: Rural-to-urban migration, discrimination experience, and health in China: Evidence from propensity score analysis
Source: PLoS One. 2020 Dec 28;15(12):e0244441. doi: 10.1371/journal.pone.0244441 (PMC7769422; doi:10.1371/journal.pone.0244441)
Supplement: S2 Table — (DOCX) [file pone.0244441.s005.docx]

S2 Table. Results of sensitivity analysis via regressions using different calipers in PSM

|  | Discrimination experience | Self-reported health ^a^ | Psychological distress | Physical discomfort ^a^ |
| --- | --- | --- | --- | --- |
| Caliper=0.25*SD, N=1218 | 0.768^***^ | 1.069 | 0.516** | 1.169 |
|  | (0.163) | (0.176) | (0.194) | (0.172) |
| Caliper=0.05, N=1,212 | 0.748^***^ | 1.075 | 0.498^*^ | 1.154 |
|  | (0.164) | (0.177) | (0.195) | (0.170) |
| Caliper=0.1, N=1,214 | 0.758^***^ | 1.088 | 0.483^*^ | 1.163 |
|  | (0.164) | (0.180) | (0.194) | (0.171) |
| Caliper=0.5, N=1,218 | 0.763^***^ | 1.071 | 0.511^**^ | 1.164 |
|  | (0.163) | (0.176) | (0.194) | (0.171) |

Note: ^a^ Exponentiated coefficients; Standard errors in parentheses.

! *p* < 0.1, * *p* < 0.05, ** *p* < 0.01, *** *p* < 0.001.
