# Supplementary material for: Rural-to-urban migration, discrimination experience, and health in China: Evidence from propensity score analysis
Source: PLoS One. 2020 Dec 28;15(12):e0244441. doi: 10.1371/journal.pone.0244441 (PMC7769422; doi:10.1371/journal.pone.0244441)
Supplement: S3 Table — (DOCX) [file pone.0244441.s006.docx]

S3 Table. Results of sensitivity analysis by mediation analysis using different calipers in PSM

|  | | **Natural indirect effect** | | |  | **Total effect** |
| --- | --- | --- | --- | --- | --- | --- |
|  | | Self-reported health ^a^ | Psychological distress | Physical discomfort ^a^ |  | Psychological distress |
| Caliper=0.25*SD, N=1218 | coef | **0.926** | **0.258** | **1.096** |  | **0.516** |
|  | BC | (0.868 0.969) ^b^ | (0.152 0.409) | (1.042 1.171) |  | (0.114 0.895) |
| Caliper=0.05, N=1,212 | coef | **0.929** | **0.250** | **1.094** |  | **0.498** |
|  | BC | (0.874 0.977) ^c^ | (0.139  0.381) | (1.046 1.171) |  | (0.108 0.874) |
| Caliper=0.1, N=1,214 | coef | **0.926** | **0.254** | **1.096** |  | **0.483** |
|  | BC | (0.866 0.970) ^d^ | (0.144 0.387) | (1.047 1.164) |  | (0.046 0.831) |
| Caliper=0.5, N=1,218 | coef | **0.927** | **0.255** | **1.095** |  | **0.511** |
|  | BC | (0.862 0.970) ^e^ | (0.151 0.409) | (1.047 1.165) |  | (0.158 0.899) |

Note: BC = bias-corrected confidence interval; Coefficients in bold are significant.

^a^ Odds ratio. ^b^ One or more parameters could not be estimated in 9 bootstrap replicates; standard-error estimates include only complete replications. ^c^ One or more parameters could not be estimated in 4 bootstrap replicates; standard-error estimates include only complete replications. ^d^ One or more parameters could not be estimated in 7 bootstrap replicates; standard-error estimates include only complete replications. ^e^ One or more parameters could not be estimated in 3 bootstrap replicates; standard-error estimates include only complete replications.
